# Back to the Basics: Usefulness of Naturally Aged Mouse Models and Immunohistochemical and Quantitative Morphologic Methods in Studying Mechanisms of Lung Aging and Associated Diseases

**DOI:** 10.3390/biomedicines11072075

**Published:** 2023-07-24

**Authors:** Gilberto Jaramillo-Rangel, María-de-Lourdes Chávez-Briones, Adriana Ancer-Arellano, Ivett Miranda-Maldonado, Marta Ortega-Martínez

**Affiliations:** Department of Pathology, School of Medicine, Autonomous University of Nuevo León, Monterrey 64460, Mexico; gilberto.jaramillorn@uanl.edu.mx (G.J.-R.); mdlourdes.chavezbrn@uanl.edu.mx (M.-d.-L.C.-B.); adar7035@gmail.com (A.A.-A.); ivettmiranda77@gmail.com (I.M.-M.)

**Keywords:** lung, aging, mouse model, immunohistochemistry, quantitative morphology, lung cancer, chronic obstructive pulmonary disease (COPD), infectious lung diseases

## Abstract

Aging-related molecular and cellular alterations in the lung contribute to an increased susceptibility of the elderly to devastating diseases. Although the study of the aging process in the lung may benefit from the use of genetically modified mouse models and omics techniques, these approaches are still not available to most researchers and produce complex results. In this article, we review works that used naturally aged mouse models, together with immunohistochemistry (IHC) and quantitative morphologic (QM) methods in the study of the mechanisms of the aging process in the lung and its most commonly associated disorders: cancer, chronic obstructive pulmonary disease (COPD), and infectious diseases. The advantage of using naturally aged mice is that they present characteristics similar to those observed in human aging. The advantage of using IHC and QM methods lies in their simplicity, economic accessibility, and easy interpretation, in addition to the fact that they provide extremely important information. The study of the aging process in the lung and its associated diseases could allow the design of appropriate therapeutic strategies, which is extremely important considering that life expectancy and the number of elderly people continue to increase considerably worldwide.

## 1. Introduction

Aging can be defined as the time-dependent functional decline that affects most living organisms [1]. During the late 20th and early 21st centuries, a shift in global demographics toward older ages became increasingly evident. Life expectancy increased from approximately 50 years in the 1900s to over 80 years at present. The elderly population (over age 65) is expected to exceed 1.5 billion by 2050 [2,3].

Advancing age is associated with a loss of reparative and regenerative capacities in various organs and an increase in the risk for chronic diseases [3]. One of the organs that most deteriorates with aging is the lung. The best-known change that occurs in the lung as it ages is alveolar enlargement, which results from the reconstruction of its extracellular matrix (ECM) [4]. Likewise, senile lung is characterized by inflammation, obstruction, and epithelial cell injury of bronchi and bronchioles [5,6]. Structural changes lead to physiological changes, including increased residual volume and functional residual capacity, a decreased static elastic recoil, and a reduced forced expiratory volume in one second [7]. In addition, the reduction in the specific homeostatic immune activities of the lungs facilitates the entry of pathogens into the lower respiratory tract [8]. Aging-related molecular and cellular aberrations in the lung contribute to an increased susceptibility of the elderly to devastating diseases, such as chronic obstructive pulmonary disease (COPD), cancer, idiopathic pulmonary fibrosis (IPF), and asthma, and leads to an increased susceptibility to environmental and infectious agents [7,8,9].

In humans, it is difficult to study age-related changes in the lung because the changes occur over a period of more than 40 years. Furthermore, it is difficult to distinguish a pure effect of age from that caused by pathological agents, because the lung is continually exposed to the environment. Therefore, animal models are required to study age-related pulmonary disorders so that therapeutic agents can be developed [10]. The mouse is the most prevalently used animal model for translational research in lung aging-related disorders. This is largely due to the high similarity between mice and humans in terms of genetic background and physiological structure. Furthermore, the mouse has, among other advantages, relatively low maintenance costs, high breeding efficiency, and short lifespan [11,12]. Mouse aging models fall into two categories: accelerated aging models (genetically modified) and natural aging models. While the formers have advantages such as a short modeling time and relatively controllable aging effect, they often exhibit characteristics not seen in normal aging. Although research with naturally aging mice would seem more time consuming, these mice develop many phenotypes similar to those observed in normal human aging [12,13]. Considering that the average lifespan of laboratory mice is about 24 months, whereas the life expectancy of humans globally is about 80 years, it has been calculated that one human year is almost equivalent to 9 mice days when correlating their entire lifespan [14].

In addition to an animal model, appropriate experimental methodologies are required to study the aging process. Thanks to the use of “omics” technologies (genomics, transcriptomics, proteomics, metabolomics, and increasingly other omics), together with advanced methods in biostatistics, computational biology, and bioinformatics, scientists are developing a new understanding of the cellular and molecular bases of diseases, shedding light on the resolution of complicated biomedical problems. Omics technologies have evolved from conventional methods, which they seem to replace. However, practical considerations, including cost, ethical, and informatics issues, remain a significant barrier to the widespread adoption of omics in medicine. Furthermore, it has been suggested that advances based on omics technologies will make only modest contributions to patient care [15,16,17,18].

**Figure 1:**
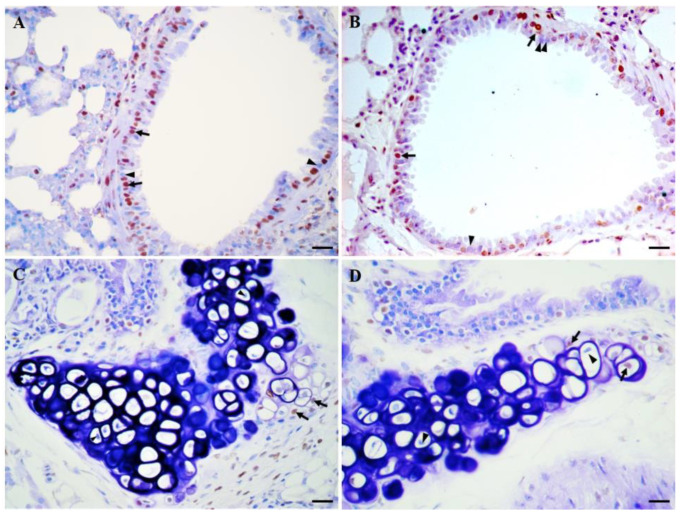
Detection of proliferating and apoptotic cells in a lung of a 6-month-old CD1 mouse by immunohistochemistry (IHC): (**A**) Immunostaining of the proliferating cell nuclear antigen (PCNA) in cells of bronchiolar epithelium (brown nucleus cells, arrows). Compare with PCNA-negative cells (arrowheads); (**B**) Apoptosis detection in cells of bronchiolar epithelium by in situ end-labeling of fragmented DNA using the TUNEL (Terminal Transferase dUTP Nick End Labeling) assay. Apoptotic cells: brown nucleus, arrows. TUNEL-negative cells: arrowheads; (**C**) Detection of proliferating cells by immunostaining of PCNA in lung cartilage (brown nucleus cells, arrows). Non-proliferating cells are pointed by arrowheads; (**D**) Apoptosis detection in cells of lung cartilage (brown nucleus cells, arrows). Compare with TUNEL-negative cells (arrowheads). Tissues were formalin-fixed and paraffin-embedded. The immunoreactivities were visualized with diaminobenzidine (DAB) and counterstaining was carried out with methyl green. Scale bar 20 µm.

Immunohistochemistry (IHC) is a technique widely used in research, more traditional than omics methodologies, but highly effective. The fundamental concept of IHC is the demonstration of antigens within tissue sections by their binding with specific antibodies. Antigen–antibody binding is demonstrated with a colored reaction visible by light or fluorescent microscopy. The advantage of IHC over other methods is the ability to visualize individual molecules and cells in nondisaggregated tissue, which makes it possible to examine important biochemical phenomena in their real context, thus augmenting and improving the information obtained [19] (Figure 1). Extremely sensitive IHC methods can detect one or multiple antigens simultaneously, or can examine hundreds of tissues in the same section for the presence of a particular antigen using tissue microarray technology. In some cases, IHC is considered the gold standard against which other techniques should be compared [20].

**Figure 2:**
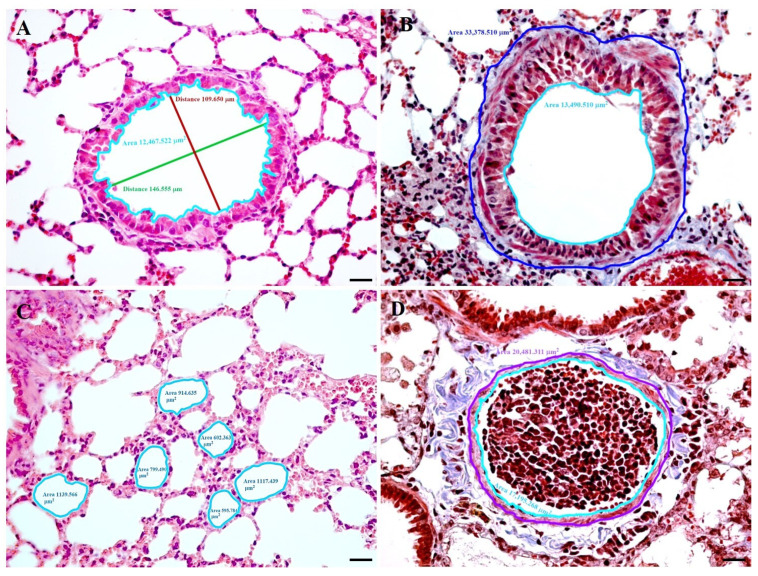
Photomicrographs showing examples of parameters that can be quantitative assessed in the lung: (**A**) The perimeter of the bronchiolar lumen (light blue line) can be calculated using the formula for the perimeter of an ellipse, in which the length of the short (red line) and long (green line) axes of the ellipse (the lumen) are considered; (**B**) Analysis of the lumen area and the total area of a bronchiole. The lumen area is the surface bounded by the apical limit of the respiratory epithelium (light blue line). The total area is the surface delimited by the outer limit of the adventitia layer (blue line); (**C**) Quantification of the alveolar area. The measured area is the surface delimited by the light blue line; (**D**) Analysis of the lumen area and the muscular layer area of a bronchiolar arteriole. The lumen area is the surface bounded by the endothelium (light blue line). The area of the muscular layer is obtained by subtracting the lumen area from the surface delimited by the outer limit of the muscular layer (purple line). Lungs of a 6-month-old CD1 mouse. Tissues were formalin-fixed and paraffin-embedded. Sections were stained with hematoxylin and eosin (**A**,**C**) or Masson trichrome technique (**B**,**D**). Scale bar 20 µm.

Despite enormous advances in investigative techniques, biomedical research requires quantitative approaches to better understand questions concerning alterations of cellular organelles, cells, and tissues, as well as to obtain a better correlation between morphology and function. To obtain quantitative information about a tissue or structure, the quantitative morphologic (QM) methods, morphometry and stereology, can be used (Figure 2). Although both terms are often used interchangeably, ‘morphometry’ is used whenever a ruler is used to measure lengths (distances). The ruler can be microscopic (stage micrometer). Stereology refers to the quantitative analysis of 3-D objects based on their 2-D appearance on cut sections. Classical stereology uses geometric probes such as frames, points, and lines for the data acquisition [21]. QM methods allow the determination of histological parameters such as volume, length, surface area, and number, in a precise, efficient, simple, and transparent manner [22,23,24]. 

From the above, it is evident that, although the study of the aging process in the lung may benefit from the use of genetically modified mouse models and omics techniques, these approaches are still not available to most researchers and produce complex results. In contrast, analysis of naturally aged mice with IHC and/or QM methods provides reliable and more direct results. In this article, we review works that used these methodologies, along with others, for the study of the mechanisms of lung aging, and in the analysis of the three most common age-related lung disorders: cancer, COPD, and infectious diseases.

## 2. Studies in Mechanisms of Lung Aging

Aoshiba and Nagai [25] hypothesized that normal aging is associated with a pro-inflammatory shift in the lung. Using the cDNA microarray technique and a quantitative analysis with the reverse transcription polymerase chain reaction (RT-PCR) assay, authors showed that several inflammation-related genes were upregulated in aged Balb/c mice (24-month-old) compared to young mice (12-week-old). IHC and a count of cells showed that the lungs of the aged mice contained increased numbers of macrophages, CD4 cells, CD8 cells, and B cells. Also, Li et al. [26] hypothesized that aging up-regulates the activation of the p38 mitogen-activated protein kinase (p38 MAPK) pathway with a corresponding increase in pro-inflammatory cytokines and a decrease in antioxidant capacity. Protein levels of phospho-p38 MAPK (the activated protein) were determined using Western blot analysis in several organs of young (2-month-old) and aged (20-month-old) C57BL/6J mice. Results showed that phospho-p38 MAPK protein levels were significantly increased in the lungs of the older mice. IHC showed that epithelial cells and alveolar macrophages in lung parenchyma were the major sources of phospho-p38 MAPK positivity. The concentrations of the pro-inflammatory cytokine tumor necrosis factor-α (TNF-α), interleukin-1β (IL-1β) and interleukin-6 (IL-6) in serum, bronchoalveolar lavage fluid (BALF), and lung homogenates from mice were determined by an enzyme-linked immunosorbent assay (ELISA). The three cytokines were elevated significantly with age in lung homogenates. There were no differences with age in serum levels except for IL-6. IL-1β and IL-6 were increased notably while TNF-α was not different with age in BALF. Furthermore, the oxidant–antioxidant status was evaluated by measuring pro-oxidant malondialdehyde (MDA) levels and the activity of the reactive oxygen species (ROS) scavenging enzymes glutathione (GSH) and superoxide dismutase (SOD) in lung homogenates. For MDA, no significant differences between the two age groups were observed, whereas total GSH and SOD levels were significantly decreased with age. Taken together, results showed that p38 MAPK is activated during lung aging with a corresponding increase in pro-inflammatory cytokines and disturbances in oxidant–antioxidant status [27,28]. Data from these and related studies [29,30] support the presence of the phenomenon called “inflammaging” in the aged lung. Inflammaging is an age-dependent chronic increase in basal systemic inflammation [31,32]. Inflammaging, along with other alterations, may predispose to the development of lung diseases in aged individuals, such as malignancies, COPD, autoimmunity and infectious diseases, and interstitial pneumonia [33].

Cellular senescence is a state of irreversible proliferative arrest that presents after a discrete number of cell divisions, whose underlying cause is telomere uncapping triggered by the continuous loss of telomere sequences during cell division. A senescent phenotype can occur due to different stimuli, including injury, ROS, irradiation, and nutrient imbalances [34,35]. The impact of cellular senescence on the aging process in an organismic context is not entirely clear, mainly due to the paucity of data on the frequency of senescent cells in tissues during the aging process. Telomere uncapping, as well as DNA double-strand breaks, induces a DNA damage response characterized by the phosphorylation of Ser-139 of histone H2A.X (γ-H2A.X) adjacent to the site of DNA damage [36]. Wang et al. [37] measured frequencies of γ-H2A.X-positive cells by IHC in tissues of C57BJ6 mice of 12, 22, 36, and 42 months of age. Frequencies increased with age in lung, liver, spleen, dermis, and intestinal epithelium. These results suggested that cellular senescence might be among the possible causes of aging. On the other hand, a terminal step of senescence is extensive heterochromatinization and formation of nuclear structures designated senescence-associated heterochromatin foci (SAHF), which contain the proteins histone macro H2A (mH2A) and heterochromatin protein 1 beta (HP1β) [38]. Using IHC, Kreiling et al. [39] found elevated levels of mH2A in lung nuclei of 24-month-old C57BL⁄6 mice compared with mice of 4 months of age. The mean levels of mH2A calculated for groups of young (5 months) and old (36 months) animals showed a highly significant 1.9-fold increase in the old group. Assays for HP1β showed similar results to those observed with mH2A. Thus, the results of this study also support a role for cellular senescence in the aging mechanism of organisms. In another study, Kwon et al. [40] investigated the expression of sirtuin (SIRT)1 and SIRT3 in the lung, kidney, adipose tissue, skin, and spleens of 6-month-old and 24-month-old mice using IHC. SIRTs are evolutionarily conserved nicotinamide adenine dinucleotide (NAD)-dependent histone deacetylases that share homology with yeast Sir2, a protein that critically regulates the lifespan of yeast [41]. Compared with that in younger mice, the expression of SIRT1 in 24-month-old mice was increased in lung, kidney, and spleen tissue, while expression of SIRT3 was decreased in lung, kidney, and adipose tissue. Thus, aging is associated with altered patterns of expression of SIRT1 and SIRT3. More research is necessary to elucidate the role of these proteins in cellular senescence, aging, and lifespan in mammals.

As commented above, senile lungs are characterized by a uniform expansion of alveolar airspaces. Alveolar enlargement causes a reduction in the maximum achievable flow in the airways during the breathing cycle [4]. Calvi et al. [42] analyzed lungs from 2-, 4-, 8-, 12-, 16-, and 20-month-old DBA/2 mice using quantitative morphology and molecular methods. The QM method, Mean Linear Intercept (MLI), demonstrated a nonlinear pattern of alveolar enlargement, without any evidence of tissue destruction, which commenced at 12 months of age and progressed thereafter. Quantitative morphology also indicated a progressive reduction in the number of airway alveolar attachments with increasing age, which was accompanied by elastase activation evidenced by IHC. Also, IHC staining for nitrotyrosine, a marker of oxidative stress, revealed a progressive increase in oxidative stress from 2 months to 20 months of age. IHC determination of apoptosis using the TUNEL (Terminal Transferase dUTP Nick End Labeling) assay showed a statistically significant increase in staining between 2 and 12 months of age. Taken together, these findings indicate that aging-associated alveolar enlargement in mice develops during middle age (8–12 months approximately) and is accompanied by early oxidative stress, cell death, and elastase activation. Other methods used in this article evidenced the role of B cell activation, exuberant immunoglobulin production, local immunoglobulin deposition, and macrophage infiltration in the alveolar enlargement observed in the aged lung. 

Elliott et al. [43] analyzed MLI and respiratory system mechanics (resistance, impedance, dynamic compliance, and hysteresis) in C57BL/6 mice at 2, 6, 18, 24, and 30 months of age. MLI was relatively unchanged between 2 and 6 months of age, but thereafter it increased exponentially in relation to total lung volume. This aging-related increase in MLI demonstrated emphysematous-like changes (alveolar enlargement) with no evidence of increased collagen deposition, the latter evidenced by the Masson trichrome technique. The greatest changes in respiratory mechanics occurred between 2 and 6 months of age. Between 6 and 18 months of age these parameters followed a more gradual change, which tended to accelerate thereafter between 24 and 30 months of age. In summary, the authors of this article demonstrated nonlinear aging-related changes in lung mechanics and alveolar enlargement in C57BL/6 mice. 

The receptor for advanced glycation end products (RAGE) has a regulatory role in various cellular and molecular processes, including inflammation, cell proliferation, apoptosis, adhesion, and differentiation [44]. In order to evaluate the role of RAGE in lung development and adult lung homeostasis, Fineschi et al. [45] generated hemizygous and homozygous transgenic mice overexpressing human RAGE. Lungs of transgenic mice of different ages (4, 8, and 20 days, and 1, 6, 10, and 18 months) were analyzed by IHC for expression of RAGE, active caspase-3, Ki-67, and transforming growth factor (TGF)-β1. Apoptosis was evaluated by the TUNEL assay. Elastin was assessed with Weigert’s stain. The QM methods MLI, secondary crest counts, destructive index (DI) values, and internal surface area (ISA) were also analyzed. Moderate RAGE hyperexpression (hemizygous mice) during lung development resulted in impaired alveolar morphogenesis and led to significant changes in QM parameters such as the number of airspaces and the size of alveolar ducts. An increase in alveolar cell apoptosis and a decrease in cell proliferation were demonstrated. Alterations in the organization and deposition of elastin and in the expression of TGF-β1 were also observed. In homozygous mice, histologic changes resembling features of human bronchopulmonary dysplasia (BPD) were observed. In the adult lung, RAGE hyperexpression was associated with a persistent inflammatory state and an increased alveolar destructive index (“destructive” emphysema). Thus, the results obtained in this study demonstrated the important role of RAGE in lung development and homeostasis. 

Interestingly, changes in alveolar area and number through the aging process are species- and strain-dependent. While the human lung maintains a very constant number of alveoli as it ages [46], a decrease is observed in female rhesus macaques [47] and dogs [48], and senile rats do not show changes in this parameter [49]. Similarly, while DBA/2 [42] and C57BL/6 [43] mice show airspace enlargement during late life, BALB/cNNia mice are resistant to age-related increases in alveolar size [50]. We determined the area and number of lung alveoli in CD1 mice at 2, 6, 12, 18, and 24 months of age. We observed an increase in alveolar area and a decrease in alveolar number through the aging process [51]. Discrepancies among studies could be related to methodological aspects or to structural pulmonary characteristics that are strongly inherent to the species and strains analyzed. Novel approaches based on computational models that incorporate age-related changes in lung structure and function might provide more information in this area. Massa et al. [52] performed morphometric and mechanical measurements in C57BL6/J and Sftpd-/- mice at 8, 27, and 80 weeks of age. Sftpd-/- mice are deficient in surfactant protein D and develop progressive age-related lung pathology characterized by inflammation. The QM parameter analyzed was the radial alveolar count (RAC), which is the number of alveoli transected by a perpendicular line drawn from the center of a respiratory bronchiole to the nearest septal division or pleural margin. RAC is proportional to the number of intact tissue septa and will decrease as acinar walls undergo destruction [53,54,55]. The experimental results were incorporated into a computational model of the mouse lung to simulate changes in lung function. By incorporating experimentally measured factors into the model in a stepwise fashion, the contribution of these factors to lung function can be evaluated. In C57BL6/J mice, RAC was reduced to an equivalent extent in 27 and 80-week-old mice, and there was a significant reduction in RAC at all ages in Sftpd(-/-) when compared to C57BL6/J. We observed similar results to those described for the C57BL6/J mice when we evaluated RAC in CD1 mice at ages 2, 12, and 24 months. There was a statistically significant difference between the RAC means at 2 months and at 12 and 24 months; there was no significant difference between the means at 12 and 24 months. Thus, our findings showed that RAC decreases during aging until it plateaus [56].

Alveoli have been the most analyzed structures in morphometric and/or stereological studies related to lung aging, while other lung components, such as bronchioles and blood vessels, have received little attention. We measured the lumen and wall areas, and the total area of bronchioles in CD1 mice at 2, 6, 12, 18, and 24 months of age. We observed in bronchioles through aging an increase in total area, an increase in lumen area, and a decrease in wall area. Lung small airways are sites of dysfunction early in the course of chronic lung diseases. Alterations in bronchiolar structure observed in this study might contribute to the development of those diseases [57]. Changes in the dimensions of bronchiolar arterioles have been hypothesized as a mechanism of pulmonary diseases in the elderly [58]. We analyzed the following parameters in the bronchiolar arterioles of the mice described above: lumen area, muscular layer area, adventitia layer area, total area, and total perimeter. There were no significant differences in the dimensions of the bronchiolar arterioles among the ages analyzed. Thus, more research is necessary to assess the possible role of small blood vessels in chronic pulmonary diseases [59].

The previous paragraphs analyzed works carried out in the context of lung damage associated with aging. But does the lung have regenerative capacity? The human lung has little regenerative capacity, which, besides, is diminished after sexual maturity; in contrast, rodents maintain lung regenerative capacity well beyond this point. However, in both humans and rodents, is evident that lung tissue typically becomes less able to regenerate with aging [60,61,62]. Paxson et al. [63] measured the age-specific effects of pneumonectomy (PNX) in C57BL6 mice of 3, 9, and 24 months of age. Pulmonary function and total surface area assessed by inspiratory capacity, vital capacity, dynamic compliance, and MLI indicated a partial loss of regenerative capacity at 9 months and complete cessation at 24 months. The proliferation and apoptosis responses of alveolar epithelial type II cells (AECII) to PNX were evaluated by IHC. AECII are important precursor cells for alveolar epithelial type I cells. Results showed that cell proliferation and regenerative capacity were both dramatically diminished in aged (24 months) mice. Post-PNX gene expression analysis showed a myofibroblast signature and more alpha-smooth muscle actin (αSMA)-positive myofibroblasts in 9-month-old than 3-month-old mice. Isolated lung fibroblasts showed a significant age-dependent loss of clonogenicity. Furthermore, lung fibroblasts isolated from 9- and 17-month-old mice showed gene expression consistent with terminal differentiation. Taken together, these data demonstrate that in mice the rate of regeneration after PNX decreases at 9 months of age and continues to decrease further in older animals. Loss of fibroblast clonogenicity and progressive myofibroblastic differentiation are implicated in the age-dependent decline in the rate of lung regeneration.

Stem and progenitor cells play essential roles in the growth, homeostasis, and repair of many tissues. A number of different precursor cell populations have been identified in the lung, including AECII, which are precursor cells for alveolar epithelial type I cells (already mentioned in the last paragraph), and club cells, which differentiate to produce ciliated cells [64]. Watson et al. [65] compared the lungs of 3- and 22-month-old C57/BL6J mice with respect to morphological and quantitative characteristics, density and function of epithelial precursor populations, and epithelial gene expression profile. There was no statistically significant difference between the two ages when MLI, airspace area, and alveolar tissue density were assessed. Also, there were no significant changes in the circularity of whole bronchioles and that of their lumens, nor in the diameters of the lumen and the bronchiolar wall. In contrast, the total cross-sectional area of the bronchioles was reduced with aging due to the presence of a lower bronchiolar wall area and a trend towards a lower bronchiolar lumen area in the 22-month-old mice. IHC and long-term EdU incorporation analysis indicated that AECII density and proliferation are maintained with age, but the density of Type I cells is reduced with aging, perhaps due to reduced Type II to Type I cell differentiation. These assays also demonstrated that the density of bronchiolar club and ciliated cells is maintained with aging, but proliferation rates of club cells decrease, indicative of an overall slowdown in cellular turnover. Finally, epithelial gene expression profiling examined by microarray analysis revealed age-related changes in multiple genes, including some with roles in cell proliferation and differentiation, and in several signaling pathways, including the Insulin Growth Factor (IGF) and TGFβ pathways. According to the authors of this article, the observed findings could be useful for understanding the mechanisms underlying age-related lung diseases.

In a study related to the work described in the previous paragraph, we examined proliferating cell nuclear antigen (PCNA) by IHC, apoptosis by the TUNEL assay, and epithelial dimensions by QM methods in bronchioles of CD1 mice at 2, 6, 12, 18, and 24 months of age. The 2-month-old mice showed a significantly higher number of proliferating (PCNA+) cells when compared with mice at all other age groups. The number of apoptotic (TUNEL+) cells in mice at 24 months of age was significantly greater than in mice at all other age groups. QM analysis revealed a decrease in the total number of epithelial cells that started at 12 months of age and progressed thereafter, and reductions in both height and area of the bronchiolar epithelium in mice at 18 and 24 months of age. These changes reflect a dysregulated epithelial regeneration process in the bronchioles that might predispose to respiratory diseases in elderly subjects [66].

Nestin protein was initially identified as a marker for neural stem cells and, since then, its expression has also been shown in various prenatal and adult cells and tissues, where it might have a role in active proliferation, wound healing, and tisular regeneration [67]. On the other hand, cartilage is a highly differentiated connective tissue that forms mechanical support for soft tissues. Cartilage consists of cells (chondroblasts and chondrocytes) and ECM. Chondroblasts are actively dividing immature cells that form ECM. When chondroblasts are completely surrounded by ECM, they are called chondrocytes. The main function of the chondrocytes is to produce, maintain, and remodel the ECM of the cartilage [68,69,70]. In the lung, cartilage forms irregular plates in the intrapulmonary bronchi and smaller bronchi. The terminal and respiratory bronchioles lack such cartilaginous plates. In the past, healthy adult chondrocytes were believed to maintain a stable quiescent phenotype and resist proliferation and differentiation throughout life [71]. We analyzed lung samples from adult CD1 mice for the presence of apoptosis by TUNEL assay and PCNA and nestin expression by IHC. Apoptosis and PCNA were detected in lung chondrocytes. Serial section analysis demonstrated that cells in apoptosis differed from PCNA + cells, indicating that cell turnover was occurring. Chondrocytes were negative for nestin, but nestin + cells were observed in perivascular cells as well as in connective tissue associated with cartilage. These results indicated that cell turnover in adult lung cartilage is possible, and that it may be mediated by nestin + cells [72]. In another work, we found nestin + cells inside pulmonary cartilage plates, and dividing cells very close to these cells. This finding indicated that nestin + cells are able of differentiating into pulmonary chondrocytes in the adult, perhaps to maintain homeostasis or repair damaged tissue [73,74]. In another study, we detected nestin + cells in connective tissue and in perivascular areas that were in close proximity to the lung airway epithelium. These cells were also found among the cells lining the airway epithelium, perhaps in order to participate in its normal turnover [75]. Thus, the stem cell reported in our works could be a pluripotent cell, capable of generating various types of lung tissues, which could provide novel approaches for the therapy of devastating lung diseases. However, several studies have reported that nestin is a marker of cancer stem cells (CSCs) expressed in malignancies of various organs, including the lung. In cancer presentation and progression, nestin expression has been associated with several processes, including cell proliferation, metastasis, and angiogenesis [76]. Therefore, more research is needed to establish the true value of nestin in therapeutic strategies for the treatment of lung diseases.

## 3. Studies in Lung Cancer

Lung cancer is the second most commonly diagnosed malignant tumor and the leading cause of cancer-related death worldwide [77]. Approximately 70% of lung cancers occur in adults 65 years of age or older [78]. There are few studies that have investigated the incidence of lung cancer in naturally aged mice. Szymanska et al. [79] described neoplastic and nonneoplastic lesions in naturally aged mice from 12 inbred strains. The prevalence of lung neoplasms was significantly higher in BALB/cW and A.CA-H2^f^/W mice than in other strains. In another study, naturally aged B6;129 mice had an incidence of lung alveolar Type II cell adenoma or carcinoma of 32% for male and 20% for female [80].

The band 4.1 (4.1B) proteins are cytoskeletal proteins that are thought to play multiple roles in nuclear architecture, structural integrity of cell shape, and protein localization at the cell membrane [81]. A truncated form of 4.1B, termed Dal-1, was found in a screen to identify gene products down-regulated in adenocarcinoma of the lung [82]. To further elucidate the function of the 4.1B/Dal-1 gene in development and tumorigenesis, Yi et al. [83] generated mice deficient for this allele. Mice homozygotes for the targeted allele developed normally. They did not demonstrate an increased rate of any type of cancer, and their life span was similar to that of their wild-type littermates. Rates of cellular proliferation (assessed by IHC for Ki-67) and apoptosis (assessed by IHC for cleaved caspase 3) in lung, brain, and mammary tissues from null mice were indistinguishable from those seen with wild-type mice. Collectively, these findings indicate that the protein 4.1B/Dal-1 is not required for normal development and that it is not directly involved in the onset of tumorigenesis.

Squamous cell carcinoma (SQCA) of the lung and its precursor lesions express high levels of keratin 14 (K14) [84]. Squamous differentiation in the epithelial lining of conducting airways occurs only under pathological conditions [85]. In order to explore the role of K14 expression in the pulmonary epithelium that normally lacks both K14 expression and squamous differentiation, the human K14 (hK14) gene was constitutively expressed in mouse airway progenitor cells using a mouse Clara cell-specific promoter. Transgenic and wild-type mice were analyzed at 1–2, 3–4, 6–7, 14, and 21 months of age. Increased expression of K14, and of the molecular markers of the squamous differentiation program, involucrin, loricrin, small proline-rich protein 1A, transglutaminase 1, and cholesterol sulfotransferase 2B1, was detected in transgenic mice by IHC and RT-PCR. Transgenic mice displayed multifocal airway cell hyperplasia, squamous metaplasia, and lung tumors with increasing age. The authors of this study concluded that constitutive expression of hK14 initiates the squamous differentiation program in the lung, but fails to promote squamous maturation [86].

A mutation in codon 273 of the p53 gene resulting in an Arg to His substitution is among the most common genetic events in lung cancer. In order to further study the role of this mutation in lung tumorigenesis, Duan et al. [87] developed a line of transgenic mice expressing the human p53(273H) gene under the transcriptional control of the human surfactant protein C (SP-C) promoter. Rates of lung cancer formation in transgenic and wild-type mice were evaluated by necropsy studies performed in animals from 4 to 24 months. Given their association with p53 mutations in human lung cancer, the rates of K-ras gene mutation and p16INK4a (p16) promoter methylation were also evaluated. No significant differences in the rate of tumor development were observed between the transgenic and wild-type mice at the extreme age ranges (4–12 and 22–24 months). In contrast, transgenic mice had a significantly higher lung tumor rate during the age of 13–21 months. IHC revealed that tumors harvested from the transgenic mice were all SP-C positive, indicating that these lesions expressed alveolar type II pneumocyte differentiation. A small percentage of the lung tumor cells also expressed the Clara cell 10 (CC-10) protein, suggesting evidence of Clara cell differentiation. This expression pattern is similar to that observed in human cancers. An age-related effect was observed for K-ras codons 12 or 13 mutations, and transgenic mice older than 13 months had a significantly higher rate of p16 promoter methylation. In summary, the expression of the mutant p53(273H) in combination with other genetic and epigenetic alterations occurring after the age of 13 months in mice, induced an age-related shift in lung tumor formation.

Vascular endothelial growth factors (VEGFs) are key mediators of the formation of blood and lymphatic vessels during embryonic development and in adults [88]. Several members have been identified in the VEGF family. The complete biological role of VEGF-D has not yet been elucidated. Kärkkäinen et al. [89] produced transgenic mice expressing the mature form of human VEGF-D (hVEGF-D). Histological sections of all major organs from transgenic and wild-type mice were examined microscopically. Transgenic mice did not show major changes in lymphatic capillary density, but did show increased angiogenesis and improved muscle regeneration after injury. On aging, transgenic mice showed a high frequency of malignant tumors, mainly breast adenocarcinomas. In addition, two skin carcinomas and two lung adenocarcinomas were found. The skin and lung tumors occurred in mice older than one year of age. In one of the mice, the breast tumor metastasized into the lungs. IHC revealed hVEGF-D expression in several tumors, suggesting a pathogenic role of hVEGF-D in tumorigenesis. There was no expression of the angiogenesis markers CD31 and CD34, nor of the lymphangiogenesis marker LYVE-1 outside the vasculature in any tumor. The authors of this work concluded that in mice hVEGF-D is an angiogenic factor associated with better muscle regeneration after ischemic injury and with a higher incidence of tumor formation, mainly of the mammary gland.

The Dbl protein is a member of the guanine nucleotide exchange factors (GEFs) family. GEFs participate in the regulation of the Rho-like proteins, which in turn regulate important cellular processes ranging from gene expression, cytoskeletal remodeling, cell proliferation, and membrane trafficking [90]. In order to obtain a better understanding of the biological role of Dbl, Ognibene et al. [91] generated Dbl knock-in mice. Mutant animals were monitored over a 21-month period. Tissue specimens were collected for histological examination and IHC analysis. Dbl knock-in mice developed, with aging, a B cell lymphoproliferation that often showed features of diffuse large B cell lymphoma (DLBCL). IHC supported the diagnosis of DLBCL with a mature B cell phenotype (CD3−, K67+, CD45R/B220+). Dbl knock-in mice displayed an increased incidence of lung tumors compared to wild-type animals, which were classified as alveolar adenomas and alveolar adenocarcinomas. These data suggest a role for Dbl in DLBCL, and that Dbl is a tumor susceptibility gene in mice.

NKX2-1 (TTF1) is a transcription factor for the development and differentiation of the thyroid, ventral forebrain, and lung. In the lung, it regulates the expression of genes in airway epithelial cells and is expressed at high frequency in small cell carcinomas and adenocarcinomas. NKX2-1 is used as a pulmonary tumor marker [92]. Secretoglobin (SCGB) 3A2 is a downstream target for NKX2-1 mainly expressed in bronchial epithelial cells. Two main roles have been described for SCGB3A2 as a growth factor during lung development and an anti-inflammatory agent in the lung [93]. The expression of SCGB3A2 was reported in human lung carcinomas, suggesting its use as a tumor marker [94]. In a study, SCGB3A2 expression was compared with NKX2-1 expression in lung cancer. To fulfill this purpose, histopathological and IHC analyses for the expression of NKX2-1 and SCGB3A2 were carried out in lung tumors from aging B6;129 mice and from 10- to 16-week-old CC10TAg transgenic mice, which express SV40 large T antigen under the mouse Scgb1a1 (Clara cell-specific 10-kDa protein, CC10) gene promoter. NKX2-1 was expressed in all types of pulmonary tumors, although more focally in carcinomas. SCGB3A2 was expressed in alveolar Type II cell carcinomas and Clara cell adenocarcinomas. For comparison, human lung cancer specimens were also subjected to the IHC assays for the detection of SCGB3A2. Seventy percent of those specimens were positive for the expression of SCGB3A2. Thus, the results of this study suggest that SCGB3A2 may be a useful marker for the diagnosis of pulmonary tumors in both mice and humans [95].

Homologous recombination (HR) is a critical pathway for the repair of DNA double-strand breaks. However, excessive or aberrant HR increases the risk of genomic misalignments that can lead to cancer-promoting mutations [96]. Sukup-Jackson et al. [97] created the Rosa26 Direct Repeat (RaDR) mice, which allows the fluorescent in situ detection and quantification of recombinant cells in intact mouse organs. Kimoto et al. [98] created RaDR mice cohorts with young (7–9 weeks) or aged (8–15 months) mice. A significant increase in the proportion of recombinant cells in aged mice compared with young mice was observed. IHC staining was performed to ascertain which cell types can undergo mutagenic recombination to express the fluorescent reporter. Results indicated that both club cells and AECII can be monitored for two major drivers of cancer: de novo mutations and clonal expansion. The findings of this study indicate that mutant cells accumulate with age in the lung, which may be an important cause for the increased risk of lung cancer with age. 

## 4. Studies in COPD

COPD is characterized by airflow limitation that is not fully reversible. Changes present in the lungs of COPD patients vary, but they may include emphysema, chronic bronchitis, and mucociliary dysfunction [99,100]. In 2019, COPD was the third most common cause of death [101]. COPD is significantly more prevalent in the elderly and in smokers than in the general population. Furthermore, COPD is highly associated with up to a 4.5-fold increased risk of lung cancer [102,103].

A linkage analysis demonstrated a significant linkage of a key intermediate phenotype of COPD on chromosome 2q [104]. DeMeo et al. [105] evaluated the expression of genes within a genomic region on chromosome 2q in a microarray data set of normal mouse lung development. The SERPINE2 gene was expressed at a signal intensity >15,000; it was most highly expressed during alveogenesis and had the greatest expression change (4.5-fold) across the developmental time series. A human lung microarray data set from individuals with severe COPD and control subjects revealed that SERPINE2 expression was higher in individuals with severe emphysema than in controls. IHC showed expression of serpine2 protein in mouse and human adult lung tissue. Afterward, the association between 48 SERPINE2 single nucleotide polymorphisms (SNPs) and severe COPD was investigated by analyzing cases and controls. A significant association between COPD and 18 SNPs was observed. Case-control and family-based haplotype analyses supported similar regions of association within the gene. Linkage models including as covariates those SNPs significant in the single-SNP and haplotype analyses, revealed LOD score attenuation most markedly in a smokers-only linkage model. In summary, the results of this study led to the identification of SERPINE2 as a potential COPD susceptibility gene.

NF-E2-related factor 2 (Nrf2) is a transcription factor that regulates the expression of several antioxidant and detoxification genes. Macrophages produce many antioxidant and detoxifying enzymes regulated by Nrf2 [106]. Suzuki et al. [107] investigated age-related differences in cigarette smoke (CS)-induced Nrf2 regulation in mouse alveolar macrophages. Alveolar macrophages were collected from C57BL/6 mice (3 months and 20 months of age) and Imprinting Control Region (ICR) mice (8–10 months and 19–20 months of age). Collected cells were exposed to cigarette smoke extract (CSE) for 6 or 24 h. Nrf2 mRNA expression was temporarily down-regulated in alveolar macrophages exposed to CSE for 6 h in both age groups; the Nrf2 mRNA level was lower in older mice than in young mice. CS-induced Nrf2 up-regulation was observed in alveolar macrophages at 24 h only in young/adult mice but not in older mice independent of the strain. On Western blotting, the intensity of the band corresponding to Nrf2 was weaker in the CSE-exposed cells of the older mice than of the young mice. The expression of the Nrf2 target genes human glutamate-cysteineligase, modifier subunit (GCLM), human heme oxygenase-1 (HO-1), and human glutathione reductase (GSR) was significantly decreased in alveolar macrophages of older mice compared with those of young mice when exposed to CSE for 6 h. These findings suggest that aging affects the induction of Nrf2 and its target genes in alveolar macrophages in response to CS in mice. On the other hand, IHC assays with alveolar macrophages and lung tissue sections from humans, showed a weak staining intensity of Nrf2 in subjects with COPD. In contrast, in lung tissue obtained from lifelong nonsmokers, Nrf2 was clearly located in the cytoplasm of alveolar macrophages and bronchiolar epithelial cells, whereas Nrf2-positive cells were sparsely located within the alveolar septa. These findings indicate that, under normal conditions, Nrf2 resides in the cytoplasm bound to its negative regulators, but when cells are exposed to oxidative or xenobiotic stress, it is released and rapidly moves to the nucleus, where it activates its target genes. The authors of this study concluded that CS induces Nrf2 activation in alveolar macrophages, and that Nrf2 expression is decreased in the alveolar macrophages of older current smokers and patients with COPD.

GSH is a major antioxidant concentrated in the epithelial lining fluid (ELF) of the lung. GSH is important in modulating the release of cytokines from cells in response to proinflammatory insults, and its levels can influence both oxidative stress and inflammation, two major factors that contribute to COPD [108]. GSH levels decline in both aging and COPD, but it is unclear whether aging affects the lung’s ability to increase GSH in response to an oxidative stimulus, such as CS, or not. In a study [109], young mice (2 months old) and aged mice (8, 13, 19, and 26 months old) were exposed to either air or acute CS. BALF was obtained and centrifuged; the resulting cell-free BALF was used for GSH and TNF-α determinations, whereas cytospin slides were prepared from the resulting cell pellet for demonstration of the macrophage marker F4/80 and of the marker of inflammation nitric oxide synthase (NOS2) by IHC. Finally, lung tissue was harvested for the analysis of 8-hydroxy-2-deoxyguanosine (8OHdG), a marker of DNA oxidation. All age groups had significantly reduced ELF GSH compared with the 2-month-old mice. CS-induced BALF macrophage activation in aged mice, as was evidenced by the change in NOS2 expression: the 13-, 19-, and 26-month-old mice showed a more than threefold increase in NOS2 expression as compared with younger mice. Levels of CS-induced TNF-α and 8OHdG were elevated in the aged mice compared to 2-month-old, young mice. These data suggest that the ability to establish an ELF GSH adaptive response to CS is impaired in aged subjects and sensitizes the lung to both inflammation and oxidation, potentially contributing to the development of CS-induced COPD.

In a related study, Moriyama et al. [110] tested the hypothesis that aging increases the susceptibility to CS-induced pulmonary inflammation. Chronic inflammation in small airways is a key feature of COPD. Neutrophils are involved in tissue damage through the release of numerous mediators. Migration of activated neutrophils into the lung is triggered by chemokines, such as keratinocyte-derived chemokine (KC) and macrophage inflammatory protein (MIP)-2. Induction of these chemokines is mediated by the activation of transcription factors, such as NF-kB, and, in turn, transcription factors are activated by CS [111]. The authors compared the levels of neutrophils (assessed by IHC assays), KC, and MIP-2 in BALF, as well as the bronchiolar expression of KC and MIP-2 mRNA, after 9 days of CS exposure in 9-week-old and 69-week-old C57BL/6J mice. The older mice had a greater neutrophil influx and higher levels of bronchiolar MIP-2 and KC expression. Afterward, in order to investigate the underlying mechanisms behind these age-related differences, the same parameters were evaluated, but in response to a single CS exposure. Aged mice showed increased numbers of neutrophils in BALF when compared with young mice. In the bronchiolar epithelium of 69-week-old mice, rapid and robust up-regulation of KC and MIP-2 mRNA was observed. Increases in KC and MIP-2 mRNA were also detected in the 9-week-old mice, but to a much lesser extent. The localization of NF-kB after a single CS exposure was examined by IHC. NF-kB was prominent in both cytoplasm and nuclei in the cells lining of the terminal bronchioles in the older mice, whereas only cytoplasmic staining was detected in 9-week-old mice. These data suggest that the CS-induced NF-kB translocation into nuclei is enhanced in the bronchiolar epithelial cells of aged mice. In summary, aging increases susceptibility to CS-induced inflammation in a mouse model. Involved in this phenomenon is an upregulation of KC and MIP-2 mRNA and nuclear translocation of NF-kB in the bronchiolar epithelium.

However, there exists evidence that aging does not enhance CS-induced COPD in the mouse. Zhou et al. [112] exposed young (3 months old) and relatively old (12 months old) mice to CS for 6 months. Measures of emphysema (MLI and surface-to-volume ratio) as well as measures of small airway remodeling (airway wall thickness, wall area per unit basement membrane length, and collagen area per unit basement membrane length) were assessed in lung tissue sections. There were no differences comparing young and old animals. Inflammatory cell infiltration of macrophages and neutrophils was investigated by IHC. Macrophages increased to the same extent in young and old animals. Tissue neutrophils were also increased by CS, but to a slightly lesser extent in the 12-month animals. The marker of oxidant damage, 8-hydroxyguanosine, was analyzed by IHC. The proportion of positive airway epithelial cells was exactly the same in young and old animals. Using laser capture microdissection and RT-PCR, expression of genes relating to anti-oxidant defense, matrix production/breakdown, inflammatory response, and senescence, were examined in airways and in parenchyma. Results showed a trend to lower expression levels in older mice and a somewhat lesser response to CS in both parenchyma and airways, but the differences were usually not marked. Thus, more research is needed to find out if aging influences the effect of CS on the lung.

## 5. Studies in Infectious Diseases

Immunosenescence is defined as the impairment in both cellular and adaptive immunity as a result of age-related change. It leads to susceptibility to infection, autoimmune diseases, and other age-related diseases [113,114]. The elderly suffer from more frequent and more severe infections compared to younger individuals and tend to have poorer outcomes. Infection is the leading cause of death in one-third of people aged 65 and over, and about 50% of all community-acquired pneumonia (CAP) cases occur in adults older than this age [115]. Furthermore, older adults with chronic illnesses including COPD, heart failure, and diabetes mellitus are more susceptible to common infections and show a weaker vaccine response than those without underlying health problems [116]. For these reasons, the use of animal models of traditional and emerging infections is necessary [117,118].

Kolopp-Sarda et al. [119] analyzed lung tissue from 22 mice of various strains (C57BL/6, DBA/2, and BALB/c), ranging in age from 28 days to 12 months. Using IHC and histochemical assays, IgA plasma cells, B and T cells, and macrophages were identified and enumerated in each sample. In all strains, macrophages were the predominant population. B-cells were usually the second most numerous subset. T-cells were always present, with Lyt2+ often less numerous than L3T4+. Small lymphoid aggregates composed of B or T cells were seen in all mice, usually next to a bronchiole and its accompanying vein. Plasma cells were in smaller numbers, scattered in the lung parenchyma or associated with bronchioles. Regarding the distribution of the different types of cells according to strain and age, the most important observation consisted of a decrease with age in T cells in C57BL/6 and DBA/2 mice, while they increased in BALB/c mice. Plasma cells were less numerous in young animals and in C57BL/6 mice at all ages. The data presented in this work could be important in studies involving the pulmonary immune response in aging, and the variation that it could have between different mouse strains.

Advanced age is associated with a weak pro-inflammatory cytokine response to bacteria and diminished phagocytosis in macrophages, called age-dependent macrophage dysfunction (ADMD) [120]. ADMD results in weak nuclear factor kappa B (NFκB) and MAPK activation. Pro-inflammatory cell signaling pathways result in the activation (polyubiquitination) of TRAF6, which in turns activates NFκB and MAPK pathways. The protein A20 is the key homeostatic suppressor of TRAF6, and the expression of A20 is induced by the pro-inflammatory cytokine TNF-α [121]. Hinojosa et al. [122] tested the hypothesis that ADMD results in elevated A20 levels in aged macrophages due to inflammaging, which is an age-dependent chronic increase in basal systemic inflammation (see above). Lung sections of young (4 months), mature (12 months), and aged (21 months) C57BL/6 mice were analyzed by IHC assays to detect A20. A20 was strongly elevated within the aged lungs, mainly in epithelial cells in alveoli and bronchi, vascular endothelial cells, and alveolar macrophages. Then, in order to determine which cytokine was involved in enhanced A20 levels, J774A.1 macrophages and alveolar macrophages from young mice were exposed to TNFα or IL-6, and A20 levels were measured. In both instances, cells exposed to TNFα had increased A20, whereas those exposed to IL-6 were unchanged vs. untreated controls. Finally, in assays of coincubation of alveolar macrophages with *Streptococcus pneumoniae*, TRAF6 polyubiquitination was diminished in alveolar macrophages isolated from aged vs. young mice. Thus, these experiments showed that aging is associated with increased A20 levels in lung cells and macrophages, perhaps due to increased TNFα levels and inflammaging. This in turn contributed to the incapacity of alveolar macrophages to respond to bacterial stimulation with a pro-inflammatory cytokine response. The authors of this article concluded that elevated A20 due to TNFα partially explains the ADMD phenotype.

Pneumonia is the leading cause of infectious death among the elderly. Given the documented presence of senescent cells in aged tissues, Shivshankar et al. [123] hypothesized that cellular senescence enhances susceptibility to pneumococcal pneumonia through increased bacterial ligand expression. Levels of the pro-inflammatory cytokines IL-1α, IL-1β, IL-6, TNFα, and the C-X-C Motif Chemokine Ligand 1 (CXCL1) were determined by ELISA in lung homogenates from young (4–5 months) and aged (19–22 months) Balb/cBy mice. Aged mice had increased cytokine levels. Also, histopathology of lung sections showed that aged mice had an increased incidence of interstitial and peribronchial inflammation. An age-dependent increase in the senescence markers p16 and pRb was observed by Western blot in mouse lung samples. On the other hand, for mature (51–63 years) and elderly (64–82 years) humans, in addition to the increase in p16 and pRb, increased levels of mH2A (another senescence marker) were also observed compared to young controls (43–50 years). These findings support the concept that cellular senescence occurs in the aged lungs and acts as a source of inflammation. Western blot analyses of mouse and human lung homogenates demonstrated an age-dependent increase in the bacterial ligands Keratin 10 (K10), laminin receptor (LR), and platelet-activating factor receptor (PAFr). IHC of mouse lung sections confirmed these observations for K10 with intense staining for both the alveolar and bronchial epithelial cells of aged mice vs. young controls. Finally, young and aged animals were challenged with *S. pneumonia*. Aged mice were found to be highly susceptible to pneumococcal challenge. These findings indicate that cellular senescence impacts inflammation and infectious disease in the lungs and provides an additional mechanism for the increased incidence of pneumonia in the elderly.

*Chlamydia pneumoniae* is a causative agent of pneumonia and bronchitis, and has also been implicated in an extensive list of chronic inflammatory diseases, which include arthritis, atherosclerosis, multiple sclerosis, Alzheimer’s disease, COPD, asthma, and primary biliary cirrhosis [124,125]. Many of these diseases linked to *C. pneumoniae* are more common or more serious in aged individuals. It is estimated that 50% of middle-aged individuals are seropositive to the bacterium; this value increases to 60–70% in subjects 60 years of age and older [126]. BALB/c mice at 6 months of age (young) or 20 months of age (old) were inoculated intranasally with 5 × 10^4^ (low dose) or 5 × 10^5^ (high dose) inclusion forming units (IFU) of *C. pneumoniae*. Lungs were excised and homogenized. Extracts were then cultured on HEp-2 cell monolayers and viable *C. pneumoniae* was enumerated in each sample. At both doses, old animals cleared lung infections less frequently than young animals. Histological analysis of lung tissue revealed loss of typical lung architecture, including alveolar consolidation and inflammatory infiltrates, in all infected mice, but more prominently in aged mice. IHC analysis of lung tissue showed positive staining for *C. pneumoniae* preferentially in areas of significant consolidation and with mononuclear cell infiltration. Finally, culture and IHC assays demonstrated dissemination of *C. pneumoniae* to cardiac tissue, the brain, and olfactory bulb, which was age and dose-dependent. In summary, the results of this study suggest that infection with *C. pneumoniae* may be more severe in old animals [127].

Sendai virus (SV), a member of the Respirovirus genus, causes severe respiratory illness in mice, which resembles pneumonia caused by human ortho- and paramyxoviruses [128]. BALB/c mice, designated as young (2 months), intermediate-aged (11–13 and 17–18 months), or aged (22–24 months), were inoculated intranasally with 100 median pneumonia doses (PD_50_) of SV. Mice were examined at 6, 10, and 20 days post-inoculation. Evaluations included clinical observation, lung virus titers, IHC for SV antigen, and histopathology. Mice of the three age groups remained asymptomatic and no signs of pneumonia were noted. In aged mice, SV titers in the lung were higher, the virus persisted longer, and antibody titers to the virus were lower than those in young mice. On day 6, SV antigen localized to the bronchiolar epithelium and occasionally to the alveolar lining cells of young and old mice. The prevalence of the antigen was higher in old mice than in young mice. By day 10, the antigen was prominent in the bronchiolar and alveolar epithelium of old mice, while only traces of antigen were found in the bronchiolar epithelium of some young mice. No antigen was found in any of the age groups on day 20. Histopathological analysis revealed that SV caused transient pneumonia in young mice. Infection ceased within 10 days of inoculation and lung repair progressed by day 20. In aged mice, necrosis in the lung was more severe, whereas inflammation developed and regressed more slowly. The intermediate-aged mice had some characteristics of young mice and others of aged mice. The results indicate that the susceptibility of mice to viral pneumonia increases gradually during aging [129].

Lower respiratory infections (LRI) with respiratory syncytial virus (RSV) during early life have been linked to the development of recurrent asthma and wheezing in later life [130]. Collins et al. [131] conducted a study to determine whether long-term alterations occur in airway function or structure following RSV infection in mice. Weanling (21 days old) and adult (8 weeks old) BALB/c mice were infected with RSV or received vehicle. Respiratory system impedance was used to assess responses to iv methacholine (MCh) at 4, 8, 24, and 34 weeks post-infection. Mice infected as adults showed no alterations in airway function. Mice infected as weanlings had increased MCh responses 24 weeks post-infection, but the increased response was not present 34 weeks post-infection. Furthermore, airway wall thickness and number of alveolar attachments per airway were measured at 24 weeks post-infection. The results did not reveal any differences between RSV infected and control animals. These data do not provide support for alterations in airway function or structure being responsible for the observed relationship between RSV infection in early life and asthma in later life.

Interestingly, Giles et al. [132] raised a hypothesis in the opposite direction to that described in the previous study. These authors hypothesized that cross-protection elicited by a single antigen early in life would be long lasting and sufficient to protect individuals years after exposure. Influenza virus-like particles (VLPs) were engineered to express the hemagglutinin (HA) and genes from the 1918 influenza virus. BALB/c mice from 8 to 12 weeks of age were vaccinated with purified VLPs. Vaccinated mice were allowed to age to a final age of 20 months and tested for cross-protection against the pandemic H1N1 strain. Vaccination with the 1918 VLPs completely protected aged mice from the pandemic H1N1 virus challenge. The duration of vaccine protection was lifelong. Histopathological analysis and IHC assays using antibodies against influenza A virus, myeloperoxidase, IgA, IgG, and IgM showed that the vaccinated animals were not protected from viral replication but restricted the virus to larger airways. They did not show signs of alveolar infection, which is the most common feature of fatal human disease. Thus, lifelong immune responses did not result in decreased total viral replication, but rather restricted the anatomical location of viral replication in the elderly.

## 6. Conclusions

**Figure 3:**
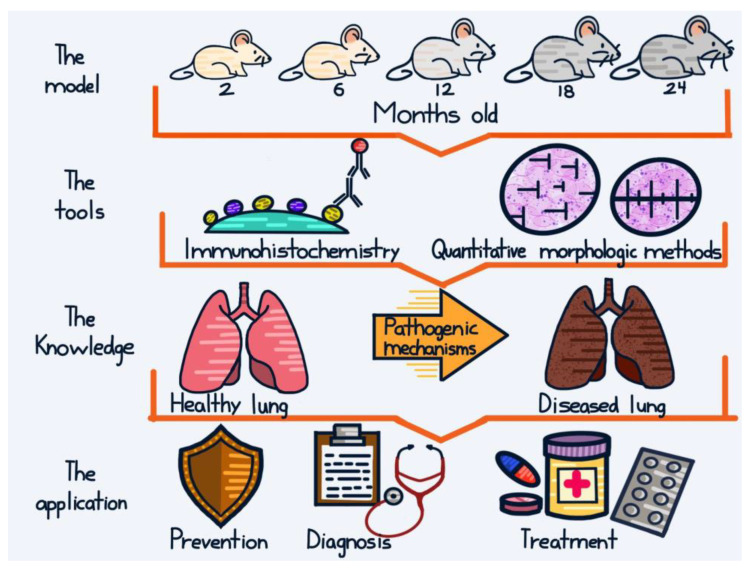
Conclusive graphical abstract. Mice of different ages, subjected to standard vivarium conditions or to stressful agents, such as cigarette smoke or infections, are analyzed with immunohistochemistry (IHC) and/or quantitative morphologic (QM) methods. IHC provides important information by allowing the demonstration of antigens (e.g., transcription factors, enzymes, receptors) in their real context. QM methods enable the determination of parameters such as volume, length, surface area, and number of diverse histological structures (e.g., cells, blood vessels, alveoli) in a precise, efficient, simple, and transparent manner. The knowledge thus obtained contributes to the elucidation of pathological mechanisms observed during lung aging, and to the design of preventive, diagnostic, and therapeutic strategies against age-related lung disorders.

The analysis of the works reviewed in this article demonstrates the usefulness of the use of naturally aged mouse models, together with the IHC and QM techniques, in the study of the mechanisms of the aging process in the lung, and its most commonly associated disorders: cancer, COPD, and infectious diseases. The information obtained could be useful in the design of appropriate preventive, diagnostics, and therapeutic strategies [133], which are urgent considering that life expectancy and the number of elderly people are increasing globally (Figure 3).

Although research with naturally aging mice would seem more time consuming, these mice develop many phenotypes similar to those observed in normal human aging. The mouse is the most prevalently used animal model for translational research in lung aging-related disorders. However, there are relevant differences between mouse and human immunology, anatomy, and physiology, which have been reviewed in the literature [134]. These differences must be taken into account when extrapolating findings from the animal model to the human disease. The advantage of IHC over other methods is the ability to visualize individual molecules and cells in their real context, augmenting and improving the information obtained. QM methods measure histological parameters in a precise, efficient, simple, and transparent manner, which allows a better correlation between morphology and function to be obtained. In the future, the application of technical improvements in these methodologies will further increase their value in biomedical research.

## Data Availability

Not applicable.

## Abbreviations

4.1B	Band 4.1 proteins
8OHdG	8-hydroxy-2-deoxyguanosine
ADMD	Age-dependent macrophage dysfunction
AECII	Alveolar epithelial type II cells
BALF	Bronchoalveolar lavage fluid
BPD	Bronchopulmonary dysplasia
CAP	Community-acquired pneumonia
CC-10	Clara cell 10 protein
COPD	Chronic obstructive pulmonary disease
CS	Cigarette smoke
CSCs	Cancer stem cells
CSE	Cigarette smoke extract
CXCL1	C-X-C motif chemokine ligand 1
DAB	Diaminobenzidine
DI	Destructive index
DLBCL	Diffuse large B cell lymphoma
ECM	Extracellular matrix
ELF	Epithelial lining fluid
ELISA	Enzyme-linked immunosorbent assay
GCLM	Glutamate-cysteineligase modifier subunit
GEFs	Guanine nucleotide exchange factors
GSH	Glutathione
GSR	Glutathione reductase
HA	Hemagglutinin
hK14	Human keratin 14
HO-1	Heme oxygenase-1
HP1β	Heterochromatin protein 1 beta
HR	Homologous recombination
hVEGF-D	Human vascular endothelial growth factor D
ICR	Imprinting control region
IFU	Inclusion forming units
IGF	Insulin growth factor
IHC	Immunohistochemistry
IL-1β	Interleukin-1β
IL-6	Interleukin-6
IPF	Idiopathic pulmonary fibrosis
ISA	Internal surface area
K10	Keratin 10
K14	Keratin 14
KC	Keratinocyte-derived chemokine
LR	Laminin receptor
LRI	Lower respiratory infections
MCh	Methacholine
MDA	Malondialdehyde
mH2A	Histone macro H2A
MIP	Macrophage inflammatory protein
MLI	Mean linear intercept
NAD	Nicotinamide adenine dinucleotide
NFκB	Nuclear factor kappa B
NOS	Nitric oxide synthase
Nrf2	NF-E2-related factor 2
p16	p16INK4a
p38 MAPK	p38 mitogen-activated protein kinase
PAFr	Platelet-activating factor receptor
PCNA	Proliferating cell nuclear antigen
PD_50_	Median pneumonia doses
PNX	Pneumonectomy
QM	Quantitative morphologic
RAC	Radial alveolar count
RaDR	Rosa26 direct repeat
RAGE	Receptor for advanced glycation end products
ROS	Reactive oxygen species
RSV	Respiratory syncytial virus
RT-PCR	Reverse transcription polymerase chain reaction
SAHF	Senescence-associated heterochromatin foci
SCGB	Secretoglobin
SIRT	Sirtuin
SNPs	Single nucleotide polymorphisms
SOD	Superoxide dismutase
SP-C	Surfactant protein C
SQCA	Squamous cell carcinoma
SV	Sendai virus
TGF	Transforming growth factor
TNF-α	Tumor necrosis factor-α
TUNEL	Terminal Transferase dUTP Nick End Labeling
VEGFs	Vascular endothelial growth factors
VLPs	Virus-like particles
αSMA	Alpha smooth muscle actin
